# Supplementary material for: A Highly Sensitive, Ultra-Durable, Eco-Friendly Ionic Skin for Human Motion Monitoring
Source: Polymers (Basel). 2022 May 6;14(9):1902. doi: 10.3390/polym14091902 (PMC9101320; doi:10.3390/polym14091902)
Supplement: Supplementary file 1 [file polymers-14-01902-s001.zip › polymers-1679476-supplementary.pdf]

polymers MDPI

Supplementary Material: A Highly Sensitive, Ultra-Durable, Eco-Friendly Ionic Skin for Human Motion Monitoring

Zhaoxin Li, Haoyan Xu, Na Jia, Yifei Li, Liangkuan Zhu and Zhuangzhi Sun

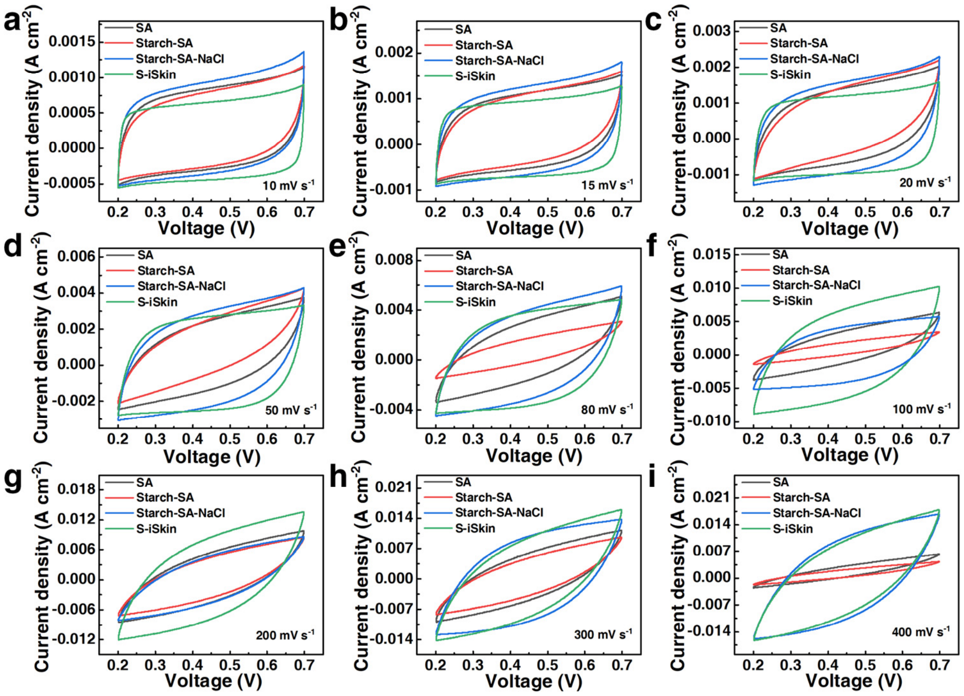

Figure S1. CV curves of the S–iSkin: (**a**) 10 mV s^{-1}, (**b**) 15 mV s^{-1}, (**c**) 20 mV s^{-1}, (**d**) 50 m V s^{-1}, (**e**) 80 mV s^{-1}, (**f**) 100 mV s^{-1}, (**g**) 200 mV s^{-1}, (**h**) 300 m V s^{-1}, (**i**) 400 m V s^{-1}.

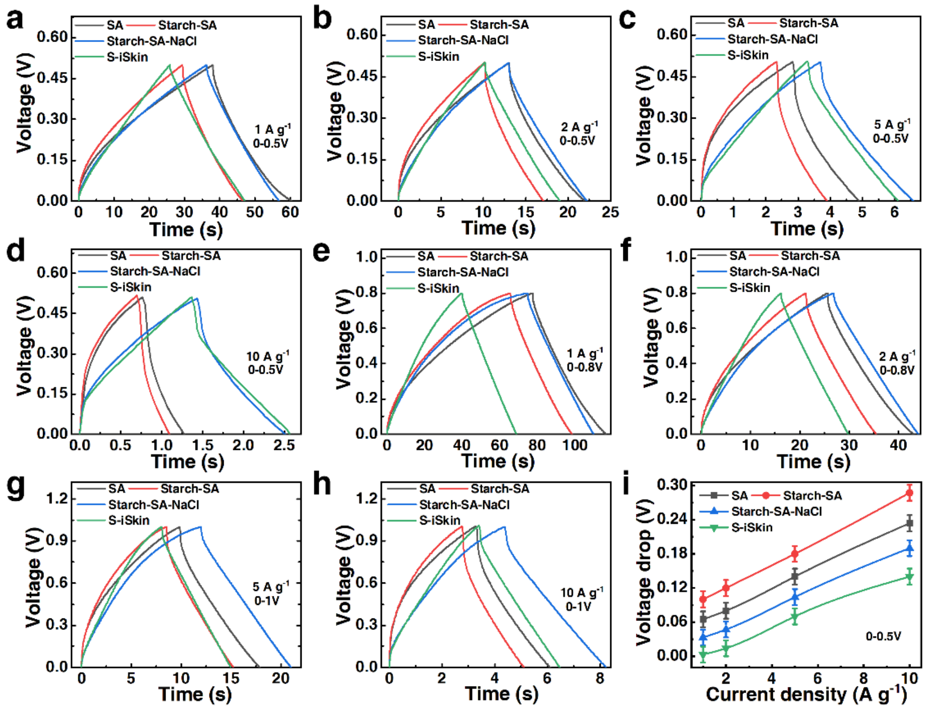

Figure S2. GCD curves of the S–iSkin under different current densities: (**a**)–(**h**) GCD curves, (**i**) Relationship curves of voltage drop varied with the current density.

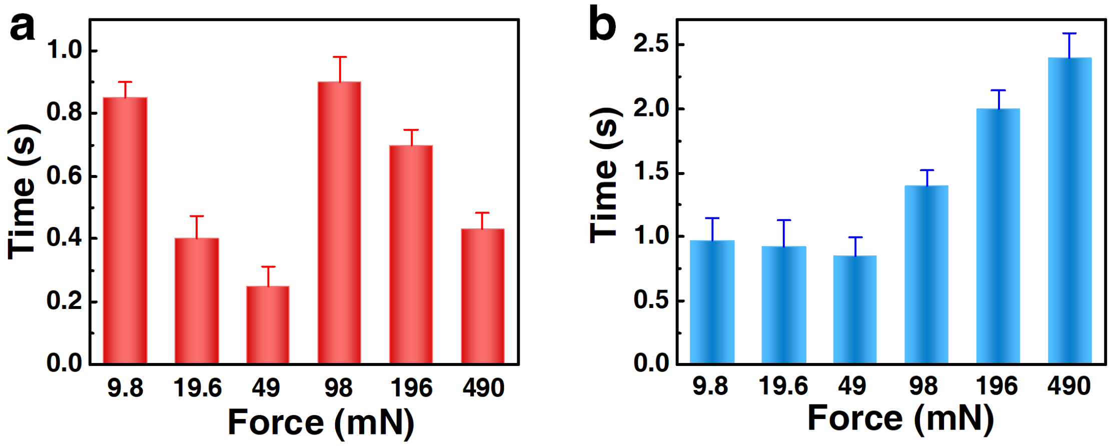

Figure S3. Response time and recovery time of the S–iSkin: (**a**) Response time and (**b**) Recovery time.

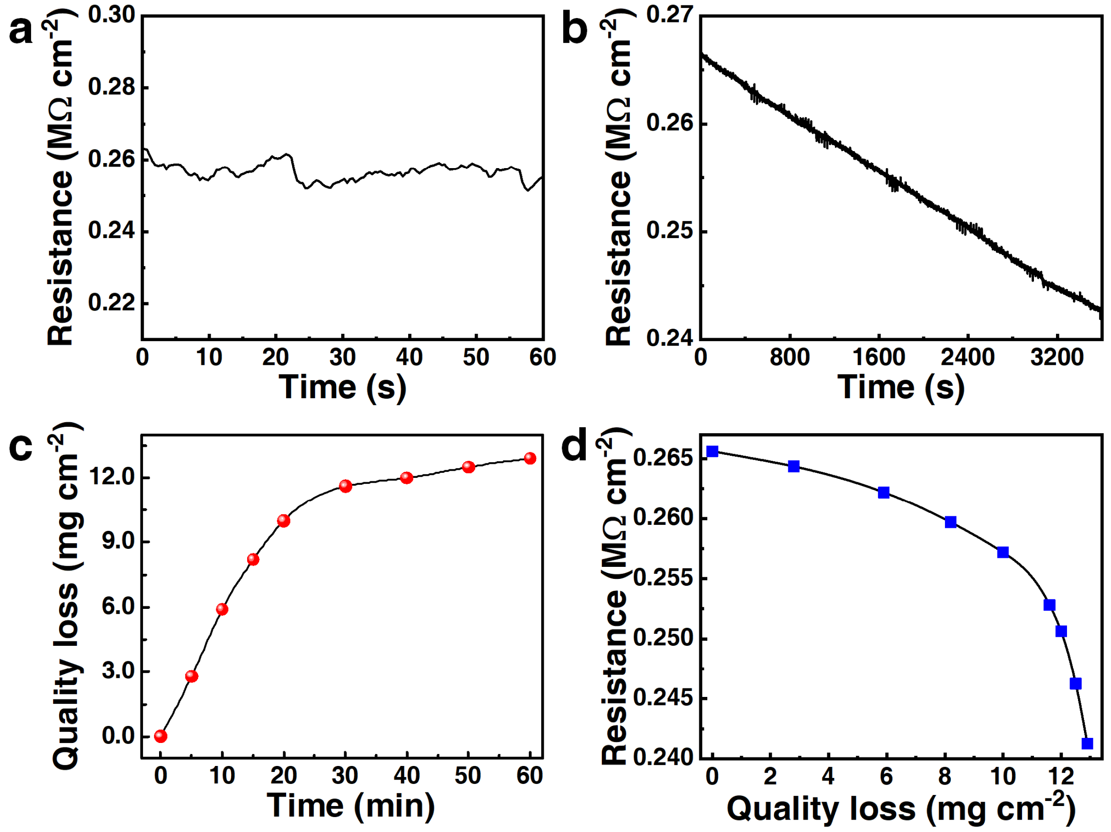

Figure S4. Variation curve of resistance and humidity of the S–iSkin: (**a**) Resistance of S–iSkin. (**b**) Variation of resistance value of the S–iSkin with evaporation of water. (**c**) The water loss rate of the S–iSkin. (**d**) The relationship between the water loss of the S–iSkin and its resistance value.

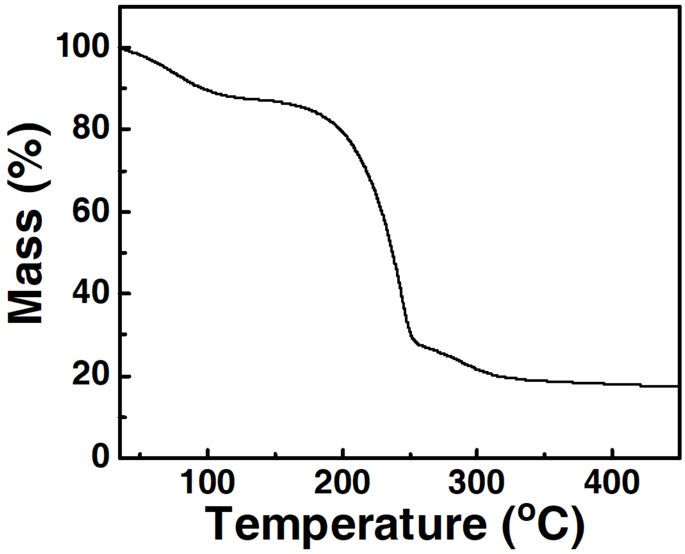

Figure S5. The thermogravimetric analysis of the S−iSkin.

Table S1. Comparison of sensitivity, response time and recovery time with other articles.

	**Sensitivity**	**Response time(ms)**	**Recovery time(ms)**
S−iSkin	52.04	15	**48**
Ref.31	0.055	60	**90**
Ref.32	0.23	27.9	**18.1**
Ref.33	74.8	36	**30**
Ref.34	64.2	720	**2820**
Ref.35	1.2	86	**101**
